# A New Ratio for Protocol Categorization

**DOI:** 10.1155/2014/389845

**Published:** 2014-03-05

**Authors:** Pierre Squara

**Affiliations:** CERIC, Clinique Ambroise Paré, 27 boulevard Victor Hugo, 92200 Neuilly-sur-Seine, France

## Abstract

The present review describes and validates a new ratio “*S*” created for matching predictability and balance between TP and TN. Validity of *S* was studied in a three-step process as follows: (i) *S* was applied to the data of a past study predicting cardiac output response to fluid bolus from response to passive leg raise (PLR); (ii) *S* was comparatively analyzed with traditional ratios by modeling different 2 ∗ 2 contingency tables in 1000 hypothetical patients; (iii) precision of *S* was compared with other ratios by computing random fluctuations in the same patients. In comparison to other ratios, *S* performs better in predicting the cardiac response to fluid bolus and supports more directly the clinical conclusions. When the proportion of false responses is high, *S* is close to the coefficient correlation (CC). When the proportion of true responses is high, *S* is the unique ratio that identifies the categorization that balances the proportion of TP and TN. The precision of *S* is close to that of CC. In conclusion, *S* should be considered for creating categories from quantitative variables; especially when matching predictability with balance between TP and TN is a concern.

## 1. Introduction

Numerous metrics and receiver operating characteristics (ROC) curves are used to test the performance of prediction methods [1]. However, these traditional tools are not, or mildly so, weighted according to the balance between TP (sign predicts event) and TN (nonsign predicts nonevent). This can be a limitation when categorizing a quantitative event with the objective of reaching occurrences (event and nonevent) of comparable probability. Indeed, predicting an event whose prevalence is close to 0 or 1 may have no clinical impact. This is especially the case when the prediction method is used to change patients' treatments and/or for including a patient in one arm of a controlled study.

For example, in patients with both severe lung injury and circulatory shock, it has been suggested to test the cardiac output response during a passive leg raise (PLR) for predicting the effects of a fluid bolus (not reversible and potentially harmful) [2–4]. Basically, we expect a linear relationship between PLR response and fluid bolus response since PLR acts like an internal transfusion. However, if we look at creating protocols driving fluid therapy in this setting, we must determine the PLR response that best discriminates relevant fluid bolus response. To achieve this, we can use an existing database and test sequentially all possible PLR response cut-off thresholds versus all possible fluid response cut-off thresholds. In this situation, the best value of traditional ratios is in two situations: (i) with high PLR thresholds (e.g., >25%) predicting high fluid bolus thresholds (e.g., >50%) and (ii) with low PLR thresholds predicting low fluid bolus thresholds. However, these two pairs of thresholds have poor practical interest. In the first case, the prevalence of high fluid response is low and most patients are classified as TN (no high PLR response predicts no high fluid bolus response). A protocol based on this would nearly never recommend fluid bolus. Conversely, in the second case, the prevalence of low fluid response is high and most patients are classified as TP (PLR response over a low threshold predicts fluid bolus response over a low threshold) and a protocol would always suggest giving fluid. Thus, actual recommendations do not result from systematic statistical analyses but from clinical and metrological (least significant change) considerations.

We present in this paper the formula of a new “*S*” ratio and show that this new ratio better matches predictability with balance between TP and TN as compared to other traditional statistics. More generally, *S* would be of interest when dichotomizing a quantitative variable in existing databases for creating protocols like cited in our example but also for decision trees and study inclusion criterion.

## 2. Methods

Measuring the quality of any categorization is a particular case of a general approach of prediction-performance assessment. These methods first define the predictor (P) and the event (E), and then determine the 2 × 2 cells matrix of TP (P predicts E), TN (non P predicts non E), FP (P predicts non E), and FN (non P predicts E).

(1)  E  Non  EPNon  P TP  FP   FN  TN 

The best method for analyzing prediction performance is to consider the whole matrix. However, it is not immediately clear if a matrix gives a proper answer to the question asked and if a given matrix is superior to another one. Different metrics have been suggested to measure the distance between P and E using a single number.

### 2.1. Traditional Ratios

Herein, two situations are clearly different.

First, when P and E are naturally binary, the most widely used ratios are as follows:

- sensitivity: (Se) = TP/(TP + FN);
- specificity: (Sp) = TN/(TN + FP);
- positive predictive value: (PPV) = TP/(TP + FP);
- negative predictive value: (NPV) = TN/(TN + FN).

The quality of the matrix may be analyzed using other ratios providing close information when P is a risk factor, a sign, or a treatment and E is a natural category like a disease or an outcome:

- risk ratio or relative risk: (RR) = (TP/(TP+FP))/(FN/(TN+FN));
- Yule's *Q* coefficient: (*Q*) = [(TP∗TN)−(FP∗FN)]/[(TP∗TN)+(FP∗FN)];
- Youden *Y* coefficient: (*Y*) = (Se + Sp − 1);
- likelihood ratio positive: (LR+) = Se/(1 − Sp);
- likelihood ratio negative: (LR−) = (1 − Se)/Sp;
- odds ratio (OR) = LR + /LR − = (TP∗TN)/(FP∗FN).

The best choice depends on the clinical context and the underlying question to be answered. For example, OR is more suitable for case-control or retrospective studies; RR is preferably used in randomized controlled trials and cohort studies [5].

In contrast, when P and E are quantitative, a binary classification requires conventional cut-off thresholds. There is consequently a different matrix for each possible P and E pair of thresholds. A series of categories can be created for each variable, based on incremental thresholds. To determine which matrix works best, the use of LRs, RR, *Q*, *Y*, and OR suffers from one to three weaknesses. (i) LRs, RR, and OR are impossible to derive when the denominator of is null and *Q* = −1 when TP or TN is null. (ii) All these ratios, except *Q* and *Y*, range from zero to infinity. This nonlinearity is poorly intuitive for quantifying practical usefulness. (iii) These ratios are marginally weighted according to the balance between TP and TN. The different methods used for assessing the matrix performance in case of dichotomized quantitative E and P have been reviewed by Baldi et al. in 2000 [1]. The best practical solution is the Pearson product-moment correlation coefficient (CC) for two binary categories, also called Matthews correlation coefficient or phi coefficient [6]. This coefficient is derived as follows:

(2) $$\begin{array}{c} \frac{\left[\left(\text{TP}*\text{TN}\right)-\left(\text{FP}*\text{FN}\right)\right]}{{\left[\left(\text{TP}+\text{FP}\right)*\left(\text{TP}+\text{FN}\right)*\left(\text{TN}+\text{FP}\right)*\left(\text{TN}+\text{FN}\right)\right]}^{1/2}}\;. \end{array}$$

It ranges from −1 to +1 and is weighed according to the proportion of observations in each category since it turns to be the square root of a chi-square (*χ* ^2^) divided by the total number of observations (*N*). However, this relationship is complex and poorly related to the balance between TP and TN when the proportion of errors (FP + FN) tends towards zero.

### 2.2. ROC Space

The ROC space is defined by the false positive rate FP/(FP + TN) = (1 − Sp, ) as *x*-axis and by the true positive rate TP/(TP + FN) = Se as *y*-axis. Each matrix represents one plot in the ROC space. Therefore, a specific ROC curve is created by a set of matrixes using a given fixed E threshold and all possible P thresholds. If E is quantitative, different curves can be created using different E thresholds. The area under ROC curves can be calculated with their confidence intervals and compared using nonparametric tests [7, 8]. The predictability increases when the area under curve becomes significantly different from 0.5, towards 1 or 0. In this latter case, P is predictive when under, rather than the above, considered threshold. ROC curves are based on a set of matrixes and cannot be compared to traditional ratios assessing one single matrix. However, the best possible matrix would yield a point in the upper left corner of the ROC space with coordinate (0, 1), obtained when FP/(FP + TN) = 0 and TP/(TP + FN) = 1, leading to an area under the curve = +1. The distance (*D*) between an observed ROC plot and this optimal value can be measured by triangulation leading to *D* = [(1−Sp)^2^+(1−Se)^2^]^1/2^. The smallest distance (*D* = 0) is obtained with 100% of TP. Similarly, with 100% of TN, the ROC plot would reach the lower right corner and the area under curve would be 0. However, a dichotomy based on these perfect predictions would not lead to a discriminative decision as seen previously. Therefore, neither ROC curves nor ROC plots are adequate answers to the issue of matching prediction and a balance between TP and TN.

### 2.3. The New *S* Ratio

After a careful literature review, it was not possible to find an adequate solution matching the predictability with a balance between TP and TN. For creating a new ratio reaching this complimentary objective, we first consider as numerator the difference between the highest possible quantity from right classifications, obtained by the product (TN∗TP), and wrong classifications, obtained by the product (FP∗FN). We standardized this quantity by using as denominator the optimal quantity obtained from the best possible balanced prediction. If *N* is the total number of observations, the best possible balanced prediction is given by TP = TN = *N*/2 and the optimal quantity is obtained by TP∗TN = *N*/2∗*N*/2 = *N* ^2^/4. Therefore,

(3) (i) S=[(TP∗TN)−(FP∗FN)](N2/4), or(ii) S=4[(TP∗TN)−(FP∗FN)]N2

The *S* ratio is symmetric, always derivable and ranges from −1 to +1. When the expected probability *p* of the event E is not exactly 0.5, *S* can be corrected by replacing 0.5 by *p* and (1 − *p*) leading to

(4)  (i) S=[(TP∗TN)−(FP∗FN)](pN∗(1−p)N), (ii) S=[(TP∗TN)−(FP∗FN)](p(1−p)N2), (iii) S=(1−p)p[(TP∗TN)−(FP∗FN)]N2.

This however does not change the linearity of the *S* ratio, just the scale and the limits, ranging from −1/(4*p*(1 − *p*)) to +1/(4*p*(1 − *p*)). In this situation, the *S* ratio becomes equivalent to Youden's *Y* coefficient, very close to the CC and to the distance from the best ROC plot. Since *S* is used in situations where we expect a balance between TP and TN, *p* approximates 0.5. In this situation, range of *S* marginally changes. For instance, if *p* = 0.4, *S* ranges from −1.04 to +1.04. Therefore, it is always suitable to use the standardized formula (3), ranging between −1 and +1.

### 2.4. The *S* Validation

For validation, first, we applied *S* to the real clinical challenge presented in the introduction. We used the data of a recently published study relating the performance of cardiac output response during PLR tests for predicting cardiac output response to 500 mL fluid bolus on 75 patients [9]. In this study, CC was used to determine the best pair of PLR and fluid bolus response cut-points. We reproduced here the complete CC table and we applied *S* to the same data to compare the two ratios.

Second, we made a complete analysis of *S* utility as compared to traditional ratios, by modeling all possible proportions in 2 ∗ 2 matrixes of TP, TN, FP, and FN, in a hypothetical population of 1000 patients. Traditional ratios were RR, LR+, LR−, OR, *χ* ^2^, *Q*, *D*, and CC, listed above plus other quantities listed by Rosner [10], including accuracy, contingency coefficient, and Equitable Threat Score. Then, we computed different proportions of total true responses (TP + TN). For each proportion of total true responses, we plotted *S* and other ratios' values when the balance between TP and TN was changing. We were therefore able to compare the impact of TP and TN balance, on each ratio, and for each level of total true responses.

Lastly, we derived the standard deviation (SD), precision (2SD/mean), and 95% confidence interval (mean  ±  2SD) of *S* by computing a random 5% fluctuation in the different 2 ∗ 2 matrixes of 1000 hypothetical observations. Further, these quantities were compared with those obtained with other ratios.

## 3. Results

### 3.1. First Step (Clinical Challenge)

Table 1(a) shows the performance of PLR tests for predicting fluid bolus responses using CC, as done in the original study, along with reproducing the complete incremental analysis. Table 1(b) depicts the *S* values applied to the same patients and the same cut-off points.

On Table 1(a), CC reaches high predictivity on the two extremities of the table. Perfect CC value = 1 is observed when PLR response ≥−15% is tested for predicting fluid bolus response ≥−15%. This is obtained from 74TP, 1TN, 0FP, and 0FN. When PLR response ≥0% is tested for predicting fluid bolus response ≥0%, CC = 0.78 is obtained from 50TP, 18TN, 0FP, and 7FN. On the other hand, when PLR response ≥25% is tested for predicting fluid bolus response ≥50%, CC = 0.7 is obtained from 1TP, 73TN, 1FP, and 0FN.

In contrast, on Table 1(b), *S* high values are concentrated in the middle of the table. The best value is reached when PLR response ≥5% is tested for predicting fluid bolus response ≥10%. This is obtained from 33TP, 32TN, 6FP, and 4FN. In addition, Table 1(b) shows that *S* isolates the highest values better than CC; in Table 1(a), 3 values are 15% close to the best score (0.78) while in Table 1(b) no *S* values are 15% close to the best score (0.73).

### 3.2. Step Two (Comparative Analysis)

**Figure 1:**
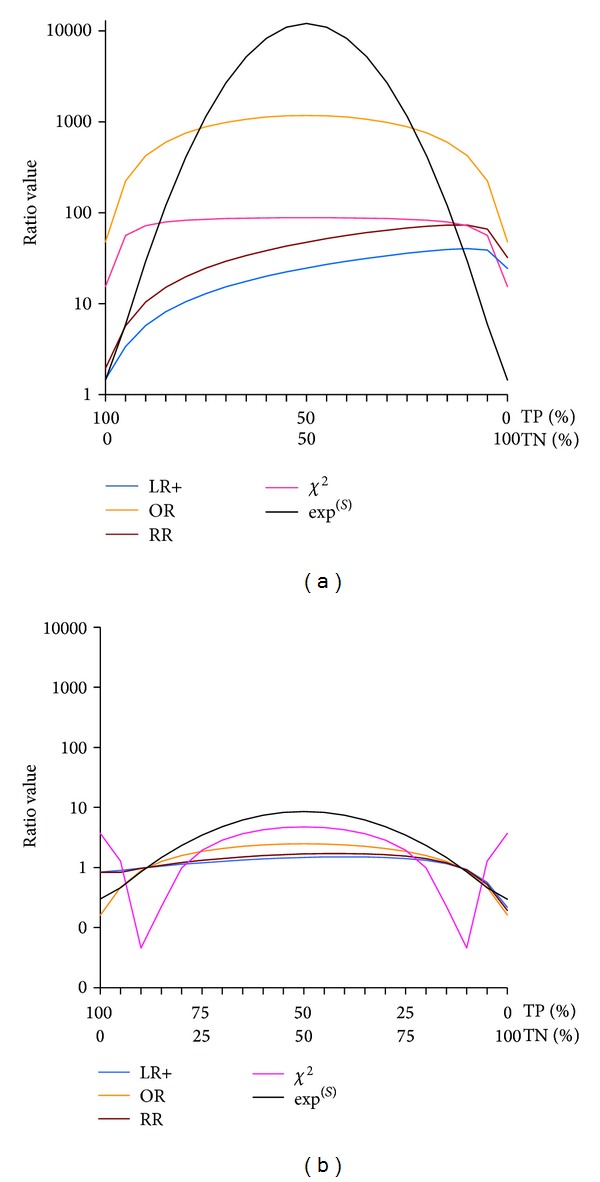
The figure shows the changes of four traditional ratios when the balance between TP and TN varies form 0–100% to 100–0%; for the same proportion of total true responses (TP + TN). The *y*-axis has log scale since these ratios range from zero to infinity. To report *S* (which is normalized from −1 to +1), we derived exp⁡^(*S*)^⁡. (a) has been computed with a high proportion of true responses (TP + TN = 97% of the total number *N*). False responses are set to FN = 1% and to FP = 2% to derive LRs, RR, and OR (not derivable if 0). (b) idem, but the proportion of total true responses is low: FP = 26% and FN = 14%; therefore, TP + TN = 60% of *N*. LR− symetric of LR+ is not represented here.

RR, LR+, LR−, OR, and *χ* ^2^ ranges from zero to infinity, so that *S* cannot be directly compared with them.  Figure 1 compares exp⁡^(*S*)^⁡ with these ratios when the proportion of TP and TN are changing and for two different levels of total true responses (97% Figure 1(a) and 60% Figure 1(b)).

**Figure 2:**
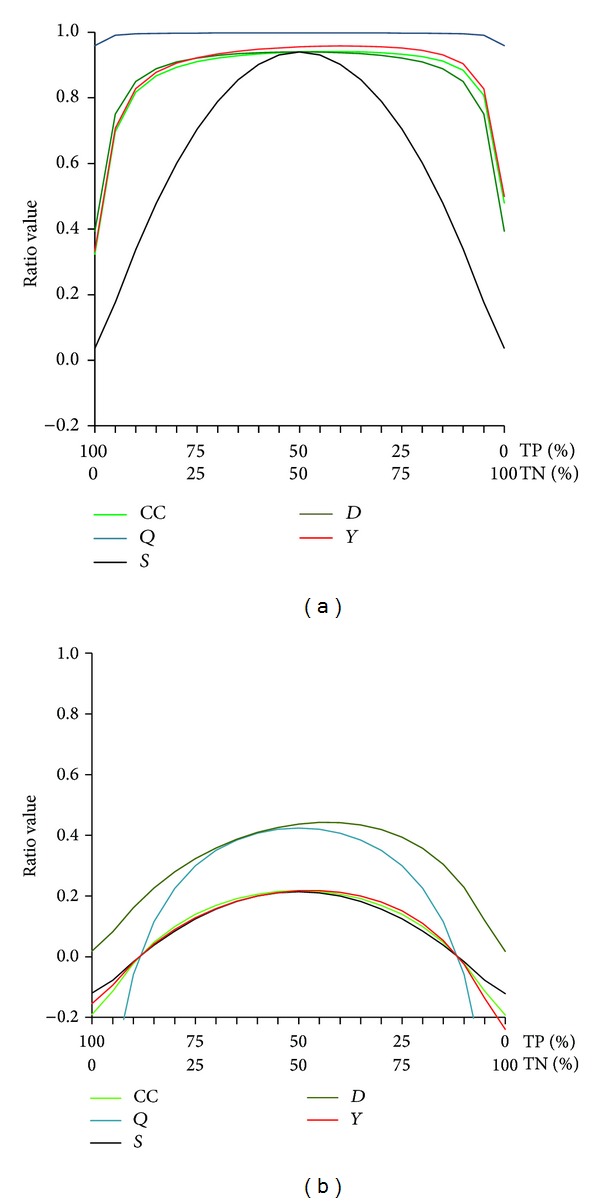
The figure represents modeling of the same proportions of true and false responses rather than those shown in Figure 1 and depicts the changes of *S* as compared to other normalized ratios: *Q*, *Y*, *D*, and CC. Therefore, the *y*-axis has standard scale.

*Q*, *Y*, *D*, and CC are normalized from −1 to +1; thus, they can be directly compared to *S* (Figure 2). The Equitable Threat Score not represented on this figure for clarity is very close to CC. All curves on Figures 1 and 2 have comparable shape than *S* curve when the predictability is poor (Figures 1(b) and 2(b)). When the predictability is good (Figures 1(a) and 2(a)), all ratios except *S* have relatively flat curve shape, indicating independence from the balance between TP and PN.

**Figure 3:**
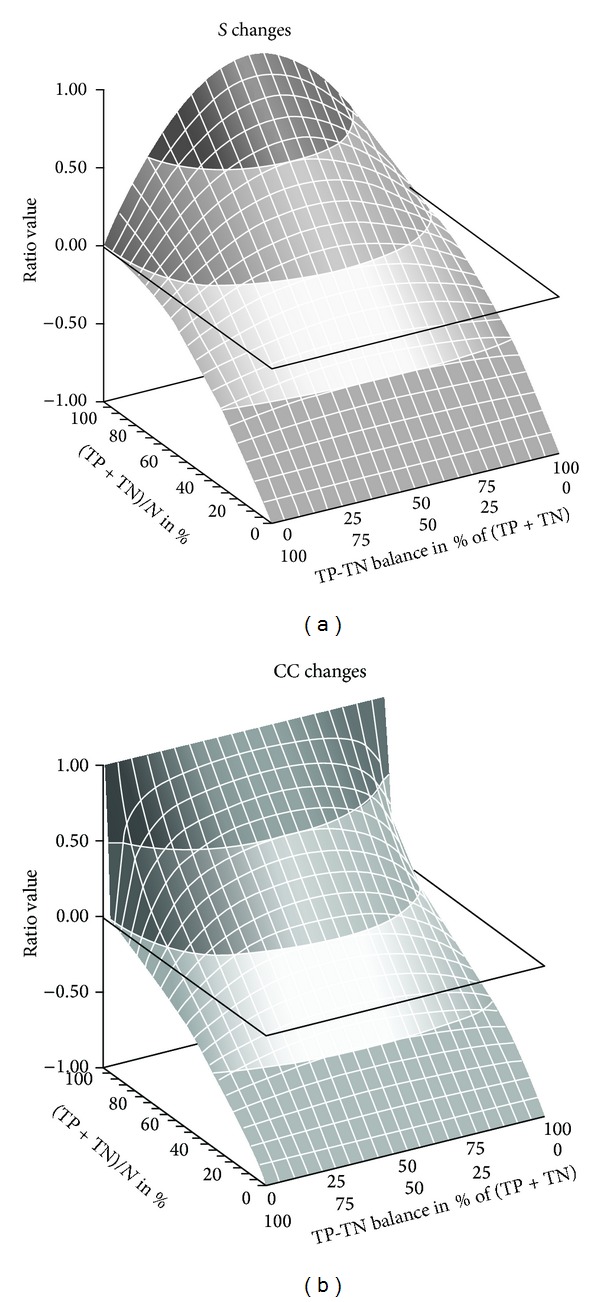
The figure shows simulating *S* and CC values according to different proportions of TP, TN, FP, and FN. On the *x*-axis are reported the different proportions of total true responses ((TP + TP)/*N*) from 100 to 0% and, therefore, the symmetrical proportion of total false responses. On the *z*-axis are reported the proportions of TP and TN for each level of total true responses. On the *y*-axis are reported the ratio values. In this figure, we have balanced the total false responses as 50% FP and 50% FN. This explains that *S* and CC have very close negative values.

**Figure 4:**
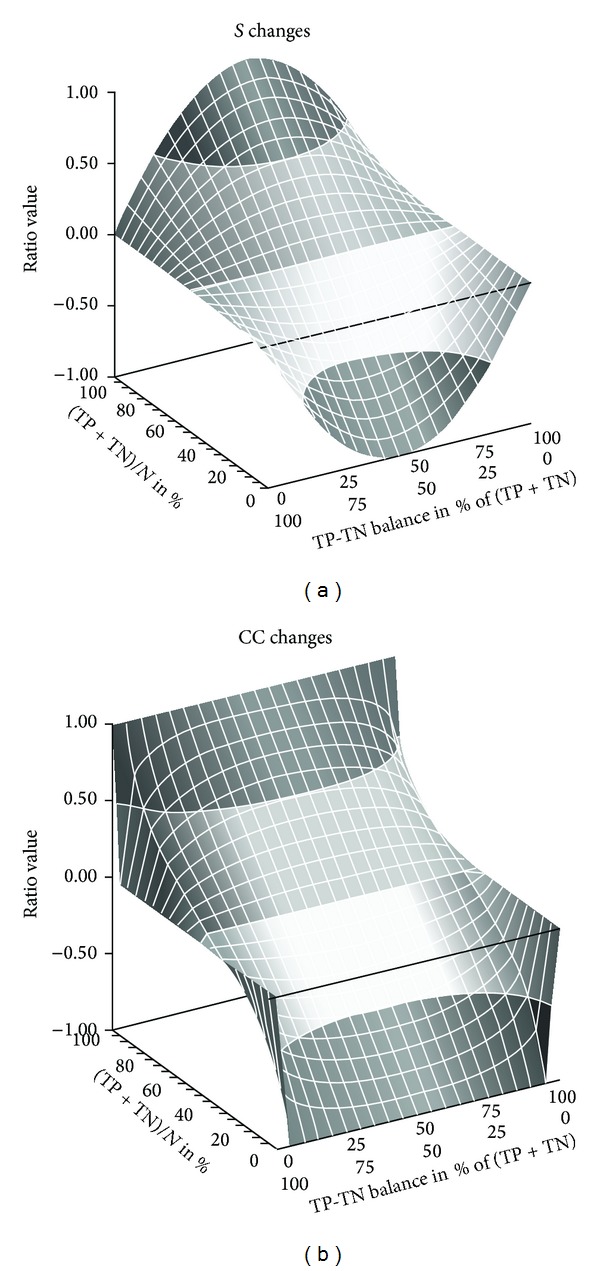
The figure idem that of Figure 3(a) but computes different balances of FP and FN as done for TN and TP. The negative values of *S* and CC are inversely symmetrical to the positive values.

Figures 1 and 2 compared *S* with other ratios using only two proportions of total true responses. A complete comparison between CC and *S* is given in Figures 3 and 4.

### 3.3. Step Three (Precision)

**Figure 5:**
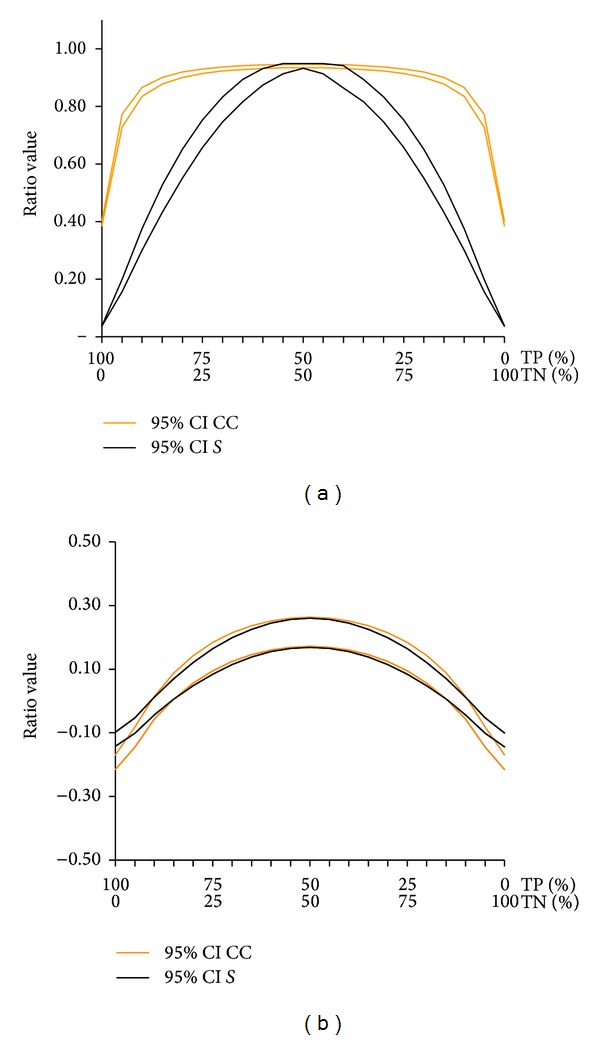
The figure compares the 95% confidence interval of *S* and those of CC. (a) and (b) represents the same proportions of true and false responses rather than those used for creating Figure 2.

The SD, precision and 95% CI of *S* is very close to these of CC, depending on the ratio value (Figure 5). For example, the average precision of on Figure 5(b) is 58% versus 56% for CC (NS). On the middle of Figure 5(a) when *S* and CC are close to 1, the precision is <1% for both ratios.

## 4. Discussion

A natural cut-off for categorizing two quantitative variables may be found when there is a clear inflexion point in their relationship. This is observed for the pressure-flow relationship in a Starling resistor [11] or the cells oxygen-supply and demand relationship [12]. In these two examples, a clear inflexion point leads to determine two clear-cut categories (pressure-flow dependency or not and oxygen-supply and demand dependency or not). Alternatively, the existence of a threshold of possible diagnostic interest may also be suspected from a clear inflexion point in a ROC curve, very close to the upper left or the lower right corner of the ROC space.

In other situations, dichotomization most often leads to loss of information, hiding the dose-response effect in most biological processes. Nevertheless, it is sometimes necessary to create conventional categories for various reasons. In this case, it is often of interest to create categories of comparable proportions and sizes; for instance, when generalizing the results of a study for creating therapeutic tests or protocols or for determining the cut-off point of an inclusion criterion of a two arms-study. Ideally, in 50% of patients, presence of an indicator will predict the event (TP) and, in 50%, the absence of this indicator will predict the nonevent (TN).

The *S* ratio described above is close to the coefficient correlation (CC) and other normalized ratios (from −1 to +1) when the predictability is poor. In contrast, when the predictive value is high, which usually is the first objective of any classification, *S* is the unique ratio weighed by the proportion of patients classified as TP and TN. Therefore, *S* appears as an interesting tool to match good predictability with two arms of comparable sizes.

The *S* ratio has few limitations. When the proportion of false response is balanced between FN and FP, *S* is lower in comparison to when FP and FN are clearly different, since the product FP ∗ FN is subtracted in the numerator of *S*. Except if homogeneity in the false responses is viewed as interesting, which seems to be a hypothetical situation, this decrease in *S* value does not indicate real loss of clinical interest. However, this effect is also observed in other ratios that share with *S* the same numerator (*Q* and CC). Moreover, this effect is mostly effective when there is a high proportion of false response thus relating to poor predictability.

In the present validation study, it was not possible to derive a specific mathematic formula for estimating the standard deviation of *S*. It would have been controversial to compare a modeled *S* confidence interval with these of other ratios based on constant chi-square boundaries or specific formulas that are often approximate. According to us, modeling a 5% random error in 1000 hypothetical patients as done presently is suitable for comparing the precision of *S* with that of other ratios. Nevertheless, determining an appropriate *S* standard deviation formula stands as future research.

The best balance between TP and TN influences decision outcome. If we create a therapeutic protocol or recommendations by generalizing the conclusion of the clinical study presented here [9], the best cut-off, identified from CC values, will lead to give fluid to all patients with positive PLR tests (we excluded negative thresholds predicting harmful fluid bolus, as done in the published results, see Table 1(a), CC = 0.78). If the studied population is representative of future patients, 76% of patients (50TP + 7FN/75) will receive fluid amongst which 67% will probably benefit (50TP/75) and 9% will not (7FN/75). Patients with a negative PLR will receive no fluid with low risk since there was no FP. In contrast, if we consider the *S*-based best dichotomization, fluid infusion will be restricted to these patients with a PLR test ≥5%, therefore to 49% of the patients (33TP + 4FN/75), among which 44% will benefit from it (33TP/75) and 5% will not (4FN/75). The other 51% of the patients will receive no fluid (32TN + 6FP/75) and, for 8% of these, it will be inappropriate (6FN/75).

In this example, use of *S*, leads to a more conservative protocol (49% of interventions instead of 76%) but with a small increase of apparent inappropriate categorization (13% versus 9%). This illustrates the fact that, by using *S*, a better balance between TP and TN may be paid by a small decrease in the absolute predictability. The final choice is a clinical decision depending on the cost and side effects of treatments as well as on the consequences of miscategorizations. In this example, we can imagine that, in a specific unit with a low incidence of lung injury, a protocol based on CC would be preferred since the risk of overfilling would be limited. However, in this type of unit, a PLR test can be viewed as unnecessary since a fluid challenge can be proposed safely. In contrast, in a unit treating acute lung injuries, the risk of overfilling would lead to the use of *S*. In their conclusions, although the researchers did not derive the *S* ratio, they recommended using that specific threshold determined by *S* (PLR  >  4% for predicting fluid response > 9%) after considering the least significant change of their measurements and the balance between risk of over treating and under treating the patients [9]. This illustrates the fact that using CC and looking at the best balance between TP and TN may provide with the same information than using *S*. However, *S* holds the advantage of allowing easier, faster, and a more reproducible approach.

The fact that *S* reached the same conclusion as clinicians in this example is not a chance. We can speculate that in this population of patients, the probability of nonoptimal filling was close to 0.5. *S* best pair of thresholds represents the highest real link including physiological and random variations. Noteworthy, the least significant change (change that have 95% chances to be real and not due to fluctuations of measurements) of the cardiac output monitoring system used here was 10%. In other words, *S* confirms that, using this specific cardiac output monitoring system, a real increase in cardiac output after fluid bolus was 10%, and it was optimally predicted by a PLR response over 5%.

We conclude that a new ratio, *S* = 4{(TP∗TN)−(FP∗FN)}/*N* ^2^, stands a consideration when creating conventional categories from quantitative variables besides when expecting a balance between true positive and true negative.

## Figures and Tables

**Table 1a:** (a) Predictability of PLR-test for fluid bolus response based on CC value

	PLR ≥−15%	PLR ≥−10%	PLR ≥−5%	PLR ≥0%	PLR ≥5%	PLR ≥10%	PLR ≥15%	PLR ≥20%	PLR ≥25%
Fluid ≥−15%	1.00*	0.49***	0.24^#^	0.17	0.12	0.08	0.04	0.03	0.03
Fluid ≥−10%	0.43***	0.41***	0.44***	0.39^#^	0.28^#^	0.18	0.09	0.06	0.06
Fluid ≥−5%	0.31^#^	0.64**	0.70**	0.54***	0.38^#^	0.25^#^	0.13	0.09	0.09
Fluid ≥0%	0.22^#^	0.45***	0.75**	0.78**	0.57***	0.41***	**0.21^#^**	0.19	0.07
Fluid ≥5%	0.15	0.31^#^	0.52***	0.71**	0.67**	0.53***	**0.27^#^**	0.27	0.09
Fluid ≥10%	0.1	0.21^#^	0.41***	0.61**	0.73**	0.65***	**0.40^#^**	0.39^#^	0.14
Fluid ≥15%	0.08	0.15	0.31^#^	0.46***	0.52***	**0.38^#^**	**0.24^#^**	0.33^#^	0.08
Fluid ≥20%	0.05	0.1	0.19	**0.28^#^**	**0.35^#^**	**0.20^#^**	**0.18**	0.22^#^	0.07
Fluid ≥25%	0.03	0.06	0.12	0.18	0.31^#^	0.19	0.19	0.25^#^	0.17
Fluid ≥30%	0.03	0.06	0.12	0.16	0.28^#^	0.25^#^	0.24^#^	0.30^#^	0.21^#^
Fluid ≥35%	0.02	0.05	0.09	0.14	0.24^#^	0.18	0.26^#^	0.25^#^	0.25^#^
Fluid ≥40%	0.02	0.03	0.05	0.08	0.13	0.18	0.28^#^	0.34^#^	0.49***
Fluid ≥45%	0.01	0.01	0.05	0.08	0.13	0.19	0.28^#^	0.34^#^	0.70**
Fluid ≥50%	imp	imp	imp	0.02	0.08	0.12	0.31^#^	0.42***	0.70**

**Table 1b:** (b) Predictability of PLR-test for fluid bolus response based on *S* value

	PLR ≥−15%	PLR ≥−10%	PLR ≥−5%	PLR ≥0%	PLR ≥5%	PLR ≥10%	PLR ≥15%	PLR ≥20%	PLR ≥25%
Fluid ≥−15%	0.05	0.05	0.05	0.04	0.03	0.02	0.01	0	0
Fluid ≥−10%	0.05	0.09	0.17	0.18	0.14	0.08	0.05	0.01	0.01
Fluid ≥−5%	0.05	0.19	0.34^#^	0.33^#^	0.25^#^	0.15	0.05	0.03	0.03
Fluid ≥0%	0.04	0.17	0.46***	0.60***	0.47***	**0.27^#^**	**0.11**	0.10	0.01
Fluid ≥5%	0.03	0.13	0.38^#^	0.59***	0.62**	0.46***	**0.17**	0.16	0.02
Fluid ≥10%	0.02	0.09	0.31^#^	0.56***	0.73**	0.62**	**0.27^#^**	0.24	0.03
Fluid ≥15%	0.02	0.07	0.21^#^	**0.39^#^**	0.51***	**0.34^#^**	**0.14**	0.19	0.02
Fluid ≥20%	0.01	0.06	0.1	**0.19**	**0.25^#^**	**0.14**	**0.12**	0.10	0.02
Fluid ≥25%	0	0.03	0.05	0.09	0.15	0.09	0.02	0.08	0.04
Fluid ≥30%	0	0.03	0.04	0.07	0.12	0.08	0.03	0.08	0.04
Fluid ≥35%	0	0.01	0.03	0.05	0.09	0.05	0.03	0.05	0.04
Fluid ≥40%	0	0	0.01	0.02	0.04	0.04	0.05	0.05	0.05
Fluid ≥45%	0	0	0.01	0.02	0.03	0.04	0.05	0.05	0.05
Fluid ≥50%	0	0	0	0.00	0.00	0.04	0.04	0.05	0.05

For better readability, values >0.8 are marked with (∗), >0.6 are marked with (∗∗), >0.4 are marked with (∗∗∗), and >0.2 are marked with (#). The bold values inside Tables 1(a) and 1(b) delimitates the data originally published (see [9]). Corresponding *S* values are also delimited in Table 1(b).

## References

[1] Baldi P, Brunak S, Chauvin Y, Andersen CAF, Nielsen H (2000). Assessing the accuracy of prediction algorithms for classification: an overview. *Bioinformatics*.

[2] Lafanechère A, Pène F, Goulenok C (2006). Changes in aortic blood flow induced by passive leg raising predict fluid responsiveness in critically ill patients. *Critical Care*.

[3] Monnet X, Rienzo M, Osman D (2006). Passive leg raising predicts fluid responsiveness in the critically ill. *Critical Care Medicine*.

[4] Billings J, Blunt I, Steventon A, Georghiou T, Lewis G, Bardsley : M (2012). Development of a predictive model to identify inpatients at risk of re-admission within 30 days of discharge (PARR-30). *BMJ Open*.

[5] Peat J, Barton B (2006). *Medical Statistics, A Guide to Data Analysis and Critical Appraisal*.

[6] Matthews BW (1975). Comparison of the predicted and observed secondary structure of T4 phage lysozyme. *Biochimica et Biophysica Acta*.

[7] Hanley JA, McNeil BJ (1982). The meaning and use of the area under a receiver operating characteristic (ROC) curve. *Radiology*.

[8] McNeil B, Hanley J (1984). Statistical approaches to the analysis of receiver operating characteristic (ROC) curves. *Medical Decision Making*.

[9] Benomar B, Ouattara A, Estagnasie P, Brusset A, Squara P (2010). Fluid responsiveness predicted by noninvasive bioreactance-based passive leg raise test. *Intensive Care Medicine*.

[10] Rosner B, Belmont RB (2006). RxC cintingency tables. *Fundamental of Biostatsitics*.

[11] Amatoury J, Kairaitis K, Wheatley JR, Bilston LE, Amis TC (2010). Onset of airflow limitation in a collapsible tube model: impact of surrounding pressure, longitudinal strain, and wall folding geometry. *Journal of Applied Physiology*.

[12] Squara P (2004). Matching total body oxygen consumption and delivery: a crucial objective?. *Intensive Care Medicine*.

